# *In Vivo* Imaging Demonstrates That *Borrelia burgdorferi ospC* Is Uniquely Expressed Temporally and Spatially throughout Experimental Infection

**DOI:** 10.1371/journal.pone.0162501

**Published:** 2016-09-09

**Authors:** Jonathan T. Skare, Dana K. Shaw, Jerome P. Trzeciakowski, Jenny A. Hyde

**Affiliations:** 1 Department of Microbial Pathogenesis and Immunology, College of Medicine, Texas A&M Health Science Center, Bryan/College Station, Texas, United States of America; 2 Department of Medical Physiology, College of Medicine, Texas A&M Health Science Center, Bryan/College Station, Texas, United States of America; University of Toledo College of Medicine and Life Sciences, UNITED STATES

## Abstract

*Borrelia burgdorferi* is a spirochetal bacterium transmitted by the *Ixodes* tick that causes Lyme disease in humans due to its ability to evade the host immune response and disseminate to multiple immunoprotective tissues. The pathogen undergoes dynamic genetic alterations important for adaptation from the tick vector to the mammalian host, but little is known regarding the changes at the transcriptional level within the distal tissues they colonize. In this study, *B*. *burgdorferi* infection and gene expression of the essential virulence determinant *ospC* was quantitatively monitored in a spatial and temporal manner utilizing reporter bioluminescent borrelial strains with *in vivo* and *ex vivo* imaging. Although expressed from a shuttle vector, the P_*ospC*_-*luc* construct exhibited a similar expression pattern relative to native *ospC*. Bacterial burden in skin, inguinal lymph node, heart, bladder and tibiotarsal joint varied between tissues and fluctuated over the course of infection possibly in response to unique cues of each microenvironment. Expression of *ospC*, when normalized for changes in bacterial load, presented unique profiles in murine tissues at different time points. The inguinal lymph node was infected with a significant *B*. *burgdorferi* burden, but showed minimal *ospC* expression. *B*. *burgdorferi* infected skin and heart induced expression of *ospC* early during infection while the bladder and tibiotarsal joint continued to display P_*ospC*_ driven luminescence throughout the 21 day time course. Localized skin borrelial burden increased dramatically in the first 96 hours following inoculation, which was not paralleled with an increase in *ospC* expression, despite the requirement of *ospC* for dermal colonization. Quantitation of bioluminescence representing *ospC* expression in individual tissues was validated by qRT-PCR of the native *ospC* transcript. Taken together, the temporal regulation of *ospC* expression in distal tissues suggests a role for this virulence determinant beyond early infection.

## Introduction

*Borrelia burgdorferi*, the etiological agent of Lyme disease, causes a multistage infection resulting in cardiac, neurologic and arthritic symptoms [1–3]. Borrelial infection is mediated through the *Ixodes* vector that transmits the pathogen to susceptible mammals, including humans, during a prolonged blood meal [4]. Infected humans may develop a painless bull’s-eye rash, known as erythema migrans, at the site of the tick bite and experience flu-like symptoms. Early antibiotic intervention is an effective treatment for clearance of infection, but when left untreated *B*. *burgdorferi* disseminates and colonizes distal immunoprotective niches resulting in severe and sustained morbidity. In 2013, the CDC reported that approximately 300,000 cases of Lyme disease occur in the United States each year, suggesting this is a significant emerging disease [5].

*B*. *burgdorferi* requires complex genetic regulation to adapt to the myriad of environmental signals it detects within the mammalian host [4,6]. *B*. *burgdorferi* alters gene expression when changes occur in temperature, pH, metals, oxygen, CO_2_, and osmotic stress; however, these environmental cues do not account for all adaptations observed from borrelial cells cultivated in implanted dialysis membrane chambers (DMC), indicating that unknown host signals also influence the response observed [7–20]. Borrelial virulence determinants needed for mammalian infection are induced through the Rrp2-RpoN-RpoS regulatory system, as well as through BosR, which is required for RpoS production [21–36]. This complex regulatory response is mediated during the tick blood meal and within the mammalian host during infection [37–42].

OspC, a well-characterized borrelial lipoprotein, is an RpoS-regulated virulence determinant and important for the establishment of early localized infection [25,35]. *B*. *burgdorferi ospC* is required for the colonization of the mammalian dermis since *ospC* mutants are cleared from the inoculation site within 48 hours after infection [25,35,43–46]. Strains in which *ospC* is constitutively expressed are not able to maintain localized or persistent infection without the ability to regulate expression similar to wild-type *B*. *burgdorferi*, suggesting that the expression of *ospC* needs to be tightly coordinated to promote the successful colonization of *B*. *burgdorferi* [47,48]. Tilly et al. demonstrated infection and dissemination of a host-adapted *ospC* mutant strain suggesting this virulence determinant is exclusively required for early stages of mammalian infection [44]. The role of *ospC* in dissemination and persistent infection in the distal tissues is not fully understood, but studies have shown a potential role for OspC beyond localized infection [49–52]. Transcripts of *ospC* increase in the heart over the course of infection, but this increase may in part be due to changes in bacterial burden [50]. Phage-display experiments showed that OspC peptides localized to the murine heart and tibiotarsal joint more so than other tissues [49], suggesting that OspC is needed at distal sites for optimal infection. Although deciphering the role of OspC during mammalian infection and its unique function has been a challenge, evidence suggests a potential role in ligand binding and/or immune evasion [52–60]. A specific example of ligand binding is the ability of OspC to bind plasminogen, although the exact binding site has yet to be identified [55,56]. Carrasco et al. showed that OspC has an anti-phagocytic role and may be involved in protecting *B*. *burgdorferi* from macrophage clearance [61]. The importance of *ospC* for infection and its presence in all borrelial strains has made it an enticing vaccine candidate, but the high degree of sequence heterogeneity and strain specific protection have historically been stumbling blocks for its further development [62–65]. Characterizing *ospC* expression *in vivo* temporally and spatially in mice would provide a modality to track the dynamic regulation of this important borrelial virulence determinant throughout experimental infection.

*In vivo* imaging technology enables the tracking of infectious pathogens in a non-invasive manner over time and locale [66–74]. The mouse model is an important tool to understand borrelial pathogenesis and genetic responses in the host environment that can not be addressed by *in vitro* modalities. To this end, *B*. *burgdorferi* was transformed with a constitutively expressed, codon optimized firefly luciferase (P_*flaB*_-*luc*) on a shuttle vector that is designed to be maintained throughout infection [75,76]. Our previous work compared the infectivity pattern of bioluminescent borrelial *bbk32* and *dbpA* mutant strains relative to wild-type *B*. *burgdorferi* [75]. From this analysis we determined that a *bbk32* mutant was incapable of establishing a strong localized infection relative to wild-type *B*. *burgdorferi*, but was able to disseminate and persist, albeit at lower levels, consistent with our prior qualitative assessment of the *bbk32* mutant [75]. This powerful approach provides the ability to track subtle, but significant, phenotypic differences seen between mutant strains during active infection in a manner not achieved by traditional endpoint studies. This technology has also been applied in other bacterial systems to evaluate changes in gene expression during the course of infection, thus providing insight regarding genetic mechanism employed in response to interaction with the host environment [77,78]. Implementing the same strategy during borrelial murine infection may provide important insight into how specific *B*. *burgdorferi* genes are regulated throughout the stages of experimental Lyme borreliosis.

In this study, we evaluated expression of *ospC* utilizing a bioluminescent reporter driven by the *ospC* promoter in a temporal and spatial context. The goal was to develop a borrelial *in vivo* reporter system to observe how *ospC* expression occurred during infection within host microenvironments of tissues following *B*. *burgdorferi* dissemination. The data presented herein is the first demonstration monitoring *B*. *burgdorferi ospC* gene expression with bioluminescence as a readout over time in live mice. Although we predicted that *ospC* expression would be limited to early stages of infection, surprisingly, *ospC* displayed unique expression patterns in various tissues at distinct time points throughout infection. The infectious load of *B*. *burgdorferi* differed between distal sites and across time points. Our results indicate that high-level *ospC* expression is not required for localized infection, but instead, low-level *ospC* expression is sufficient for colonization. Furthermore, continued *ospC* expression was observed in distal sites later in infection, suggesting a potential new role for OspC in secondary colonization or persistence.

## Materials and Methods

### Bacterial strains and plasmids

*E*. *coli* strains were grown in Lysogeny broth (LB) media under aerobic conditions at 37°C (Table 1). Concentrations of antibiotics used in *E*. *coli* for selective pressure are as follows: kanamycin, 50 μg/ml and spectinomycin, 50 μg/ml. *B*. *burgdorferi* strains were grown in BSK-II media supplemented with 6% normal rabbit serum (Pel-Freez Biologicals, Rogers, AR), referred to as complete BSKII, under conventional microaerobic conditions (1% CO_2_, 32°C) (Table 1) [79,80]. Borrelial strains were grown under antibiotic selective pressure when appropriate with kanamycin at 300 μg/ml. The Institute Biosafety Committee at Texas A&M University approved the use of infectious *B*. *burgdorferi* described in this study.

**Table 1:** Strains and Plasmids used in this study.

***B*. *burgdorferi* strains used in this study:**		
Strain	Genotype	Reference
ML23	Clonal isolate lacking lp25	[87]
ML23 pBBE22luc	Missing lp25, complemented with BBE22 and P*flaB-luc*	[75]
ML23 pJH410	Missing lp25, complemented with BBE22 and P*ospC-luc*	this study
***E*. *coli* strains used in this study:**		
Strain	Genotype	Reference
Mach-1TM-T1R	Φ80*lacZ*ΔM15 Δ*lacX*74 *hsdR* (r_k_^-^,m_k_^+^) ΔrecA1398 endA1 tonA	Life Technologies
**Plasmids used in this study:**		
Plasmid	Comments/Source/Reference	Resistance
pCR2.1 TOPO	Life Technology PCR cloning vector	kan^R^
pCR8/GW/TOPO	Life Technology PCR cloning vector	specR
pJSB161	pJD7 carrying a promoterless luc [76]	specR
pBS103	pJD7 with PospC-luc	specR
pJH409	pCR8/GW/TOPO with PospC-luc flanked by engineered PstI sites	specR
pBBE22	borrelial shuttle vector pBSV2 containing pncA (bbe22) fragment to restore infectivity in ML23 [100]	kan^R^
pJH410	pBBE22 carrying P_*ospC*_*-luc* cloned into PstI site	kan^R^
pRecA	pCR8-TOPO carrying B. burgdorferi recA [101]	specR
pβactin	pCR8-TOPO carrying murine βactin [102]	specR
pOspC	pCR2.1 carrying *ospC* from *B*. *burgdorferi*	kan^R^

### Generated constructs and modification of *B*. *burgdorferi*

A bioluminescent *ospC* reporter shuttle vector, pJH410, was generated through the PCR amplification of the native *ospC* promoter including 250 bp upstream from the ATG start codon for *ospC* and borrelial codon optimized *luc* (Table 1) [36,81]. Primers for cloning are listed in Table 2. P_*ospC*_ was amplified with NcoI restriction sites, cloned into pCR2.1 TOPO, and then transformed into Mach-I *E*. *coli* cells (Life Technologies). P_*ospC*_ was cloned into pJSB161 [76] at the NcoI site, transformed into Mach-I *E*. *coli* cells and screened for insert and orientation, resulting in pBS103. P_*ospC*_-*luc* was PCR amplified with PstI restriction sites and cloned into pCR8/GW/TOPO (Life Technologies), resulting in pJH409. The vectors were screened by restriction enzyme digest and verified through dideoxy sequencing. P_*ospC*_-*luc* was ligated into pBBE22 at the PstI to yield the final pJH410 construct. *B*. *burgdorferi* strain ML23 was made competent and transformed with pJH410 as described previously [82,83]. Transformants were selected for resistance to kanamycin and all putative isolates were screened for P_*ospC*_-*luc* shuttle vector and plasmid content by PCR followed by *in vitro* luminescence assay [75,76].

**Table 2:** Primers used in this study.

Primer	Purpose	Sequence
PospCF-NcoI	Cloning	GTATAAACGCCATGGTCTCTAATTC
PospCR-NcoI		CTTTTCCATGGATTTGTGCCTCC
PospCF-PstI	Cloning	ACGCCTGCAGGCCTGAGTATTCATTATATAAGT
lucR-PstI		ACGCCTGCAGAAGCTTTTATTATACAGC
ospCF	Cloning	GGGATCCAAAATCTAATACAAG
ospCR		GCCAAAACCGTTTAAGCCTAC
RTospCF	qRT-PCR	CGGATTCTAATGCGGTTTTACTTG
RTospCR		CAATAGCTTTAGCAGCAATTTCATCT
nTM17FrecA	qPCR	GTGGATCTATTGTATTAGATGAGGCTCTCG
nTM17RrecA		GCCAAAGTTCTGCAACATTAACACCTAAAG
qPCR-Bactin-F	qPCR	ACGCAGAGGGAAATCGTGCGTGAC
qPCR-Bactin-R1		ACGCGGGAGGAAGAGGATGCGGCAGTG

A construct for quantification of *ospC* expression was generated by amplifying a region of cp26 containing *ospC*, cloning into pCR2.1 TOPO (Life Technologies), and transforming this construct into Mach-I *E*. *coli* cells (Tables 1 & 2). The resulting plasmid was designated pOspC (Table 2).

### Western immunoblot analysis

Borrelial cells were pelleted and resolved by sodium dodecyl sulfate-polyacrylamide gel electrophoresis (SDS-PAGE) and transferred to a PVDF membrane for Western analysis as previously described [7,8,84]. Protein production was assessed using mouse monoclonal antisera to *B*. *burgdorferi* OspC (generously provided by Richard Marconi, Virginia Commonwealth University) and flagellum (Affinity BioReagents, Golden, CO) or goat polyclonal antisera to Firefly luciferase (AbCam Inc., Cambridge, MA), followed by incubation with rabbit anti-mouse IgG conjugated to horseradish peroxidase (HRP) or rabbit anti-goat IgG HRP, respectively [52,75]. The membrane bound immune complexes were visualized using the Western Lightning Chemiluminescence Reagent Plus detection system (Perkin Elmer).

### *In vitro* bioluminescence assays

*B*. *burgdorferi* was grown to mid-log phase at pH 7 or pH 8 and concentrated to 10^8^ cells/ml for ML23 pBBE22*luc* (P_*flaB*_*-luc*) and ML23 pJH410 (P_*ospC*_*-luc*). Cells were serially diluted from 10^7^ to 100 cells/ml and 100 μl of each sample was transferred to a white flat-bottom microtiter 96 well plate. Luminescence was measured using 2104 EnVision Multilabel Reader (Perkin Elmer, Inc., Waltham, MA) as previously described [75]. The different cell concentrations of each strain were treated with a final concentration of 667 μM D-luciferin (Research Products International Corp., Mt. Prospect, IL) in PBS and immediately measured for luminescence [75,69]. Cell samples for each strain from three independent cultures were measured for luminescence (photons/sec), averaged, and standard error calculated.

### *In vivo* and *ex vivo* bioluminescence studies and luminescence quantitation

Six to eight week old female Balb/c mice (Charles Rivers) were infected with 10^5^ ML23 pBBE22*luc* or ML23 pJH410 by ventral intradermal injection. Balb/c mice are the preferred strain for *in vivo* imaging due to the lack of melanin in the skin and fur, but have a distinct pathology and higher ID_50_ in response *B*. *burgdorferi* infection relative to C3H mice [85]. An inoculum dose of 10^5^ was used for this study due to maximize the measurable bioluminescence signal from P_*ospC*_*-luc* infected mice. Mice were treated with 5 mg D-luciferin dissolved in 100 μl PBS by intraperitoneal injection 10 minutes prior to imaging with an IVIS Spectrum live animal imaging system (Perkin Elmer, Waltham, MA). As a negative control for background luminescence, one infected mouse in each group did not receive D-luciferin [75]. Mice were randomly selected and *in vivo* imaging was performed 1 h and 4, 7, 10, 14 and 21 days after infection with the abovementioned borrelial strains using the methods previously described [69,82]. Luminescence was measured using 1 and 10 min exposures to obtain images for quantification and visual representation, respectively. Images were analyzed using Living Image Software from Perkin Elmer. Regions of interest (ROI) tool were selected to measure the luminescence in photons/second [p/s] from exposure images with 600–600,000 counts using an equal area of the whole body for all mice in all experiments. Background luminescence was subtracted from luminescence values for normalization. Luminescence of D-luciferin treated mice was averaged and standard error determined after normalization. All images from the 10 min exposures were treated equally when corrected for background and depicted by the radiance scale.

Housing, diet, and care of mice were under standard ABSL-2 parameters under the supervision of a Texas A&M University veterinarian. Mice were monitored daily and evaluated for general health and activity. Throughout the 21 day infection no illnesses or unexpected deaths occurred and efforts were made to minimize any discomfort to the mice. Isoflurane was utilized as an anesthetic for IVIS imaging. At designated time points mice were euthanized in accordance with guidelines of the American Veterinary Medical Association (AVMA) and as approved by the Texas A&M University Institutional Animal Care and Use Committee (IACUC).

To maximize bioluminescence during *ex vivo* imaging of infected tissues, mice were given a double bolus of D-luciferin by intradermal injection that then circulated for 10 minutes. Mice were individually sacrificed for tissues to be harvested in a timely manner to determine tissue localization of bioluminescence observed during *in vivo* imaging that measures through various tissues simultaneously. Harvested tissues were transferred to a 4 mM D-luciferin and 2 mM ATP soak for 3 minutes. *Ex vivo* bioluminescent tissues were imaged for 1 and 10 minutes, similar to *in vivo* imaging. Quantitation of tissue bioluminescence was determined by radiance (p/sec/cm^2^/sr) to account for the area difference of each tissue. Following imaging, tissues were stored in RNAlater (Life Technologies) at -80°C until isolation of total RNA. Skin samples were processed for qPCR.

### DNA and RNA extraction of *B*. *burgdorferi* from infected tissues

DNA was extracted from skin samples using Roche High Pure PCR template preparation kit as previously described [84]. RNA was extracted from infected tissue by phase separation with Trizol per manufacture instructions (ThermoFisher). RNAlater stored tissues were homogenized on ice in Trizol. Samples were incubated at room temperature for 5 minutes, then 200 μl of chloroform per ml of Trizol was added to each sample, and incubated at room temperature for 3 minutes. Phase separation occurred by centrifuging samples at 12,000 x *g* for 15 minutes at 4°C. The RNA-containing upper aqueous layer was precipitated with an equal volume of cold 100% isopropanol and washed twice with 75% EtOH to remove salt. 30 μg of total RNA was treated with 5 units of Roche recombinant DNase (RNase-free) per the manufacture instructions to remove contaminating DNA and purified by standard phenol-chloroform-isoamyl extraction followed by ethanol precipitation with 10 μg of glycogen per sample.

### Quantitative PCR and RT-PCR analysis

The Applied Biosystems ABI 7500 real time PCR system (ThermoFisher) was used to determine genomic equivalents as previously described [86]. Borrelial genomic equivalents were evaluated using primers nTM17FrecA and nTM17RrecA to *B*. *burgdorferi recA* and mouse *β-actin* copies were detected using primers Bactin_F and Bactin_R1 as previously described (Table 2). The numbers of *recA* and *β-actin* copies were calculated by establishing a C_t_ standard curve of known amount of each gene for comparison to the C_t_ values of the experimental samples. 100 ng of each experimental sample was measured in triplicate and values are displayed as copies of *B*. *burgdorferi recA* per 10^6^ mouse β-actin.

Borrelial mRNA were converted to cDNA with 3 μg DNase treated total RNA with Superscript-II Reverse Transcriptase in a 20 μl reaction as per the manufacturers instructions (ThermoFisher). Transcripts of *ospC* were quantified using PowerUp Sybr Mastermix using 2.5 μl cDNA and primers listed in Table 2 [7]. The numbers of transcript copies were calculated by establishing a C_t_ standard curve of known amount *ospC* for comparison to the C_t_ values of the experimental samples. All samples were measured in triplicate and values are displayed in copies of *ospC* for the entire tissue.

### Statistical analyses

One-way analysis of variance (ANOVA) was used to evaluate the significance of the main effects and interactions among variables to determine statistical significance in P_*flaB*_*-luc* or as represented by photons/sec (*in vivo*) or radiance (*ex vivo*) through IVIS imaging using GraphPad Prism (GraphPad Software, Inc, La Jolla, CA). Bioluminescence value for each individual mouse or tissue in a group and time point was utilized for ANOVA analysis to determine significant changes over the course of the 21 day infection. Mann-Whitney one-tail test compared medians of two groups to determine significance. P-values less than 0.05 were considered significant for all statistical analyses. Correlation between P_*ospC*_*-luc* radiance and qRT-PCR native *ospC* was also calculated using GraphPad Prism. In permutation tests, we computed the sampling distributions, also referred to as random distribution, of the differences in P_*ospC*_*-luc*/P_*flaB*_*-luc* radiance ratios across time or between set time points. To calculate these distributions, individual P_*ospC*_*-luc* and P_*flaB*_*-luc* radiance values were randomly shuffled (10,000 times) to form new ratios that were then differenced between time points. The distribution these random differences estimates the null hypothesis of no significant change in the ratio with time. The difference in the ratio actually observed was compared against this null distribution to determine the probability that a difference as great as, or greater than, the observed difference could occur by chance. Permutation tests were performed using R, a freely available language and environment for statistical computing and graphics (ver. 3.2.3; https://cran.r-project.org/).

### Ethics statement

Animal experiments were performed in accordance to National Institute of Health (NIH) Guide for Care and Use of Laboratory Animals. Animal experiments also followed the guidelines of the Association for Assessment and Accreditation of Laboratory Animal Care (AAALAC). Approval for animal procedures was given by the Texas A&M University Institutional Animal Care and Use Committee (IACUC). Mice were euthanized in manner that conforms to the guidelines put forth by the AVMA and was approved by the Texas A&M University IACUC.

## Results

### Evaluation of the *ospC* reporter in cultivated *B*. *burgdorferi*

**Fig 1:**
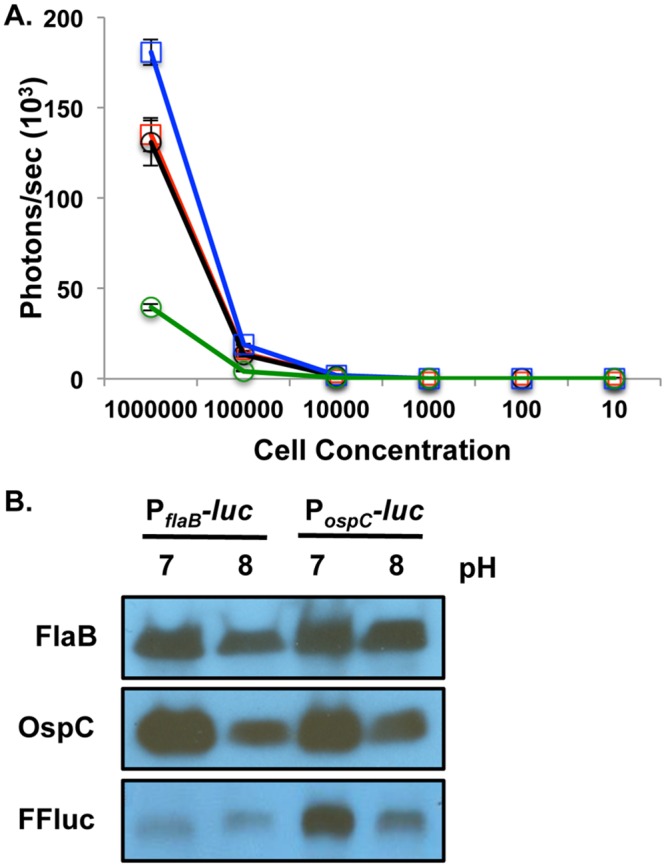
Characterization of a bioluminescent *B*. *burgdorferi* P_*ospC*_ reporter strain. The response of borrelial *ospC* reporter strain to pH was assessed to determine the validity of P_*ospC*_*-luc* relative to native OspC production. P_*flaB*_*-luc* and P_*ospC*_*-luc B*. *burgdorferi* strains were grown at pH 7 and pH 8 to mid-log phase to assess *in vitro* luminescence assay and protein production via Western blot analysis. (A) P_*flaB*_*-luc* and P_*ospC*_*-luc* cultures were serial diluted from 10^6^ to 10 cells, treated with D-luciferin, and luminescence was measured in photons/sec. P_*flaB*_*-luc* pH 7 (red squares) and P_*flaB*_*-luc* pH 8 (black circles) did not differ in bioluminescence. P_*ospC*_*-luc* pH 7 (blue squares) induces greater luminescence relative to P_*ospC*_*-luc* pH 8 (green circles). Values represent three independent cultures that were normalized to background and averaged. Error bars represent standard error. (B) Differential protein production of Luc in P_*ospC*_*-luc* reporter strain in response to pH reflects changes observed for the native OspC in P_*flaB*_*-luc* and P_*ospC*_*-luc*. Cell lysates of P_*flaB*_*-luc* and P_*ospC*_*-luc* at pH 7 or pH 8 were immunoblotted and probed with anti-sera against OspC, FFluc and FlaB that served as a loading control.

Previous studies utilized a codon optimized firefly luciferase (*luc)* in *B*. *burgdorferi* as an *in vitro* transcriptional reporter and for detection of live *B*. *burgdorferi* during experimental infection [75,76]. The high level of sensitivity observed *in vivo* with the constitutively expressed *luc* gene indicated that the system might be adapted to characterize the temporal and spatial expression patterns of specific borrelial genes in the mammalian model. To test this, we chose to track the expression of *ospC* due to its importance in the infectious process of *B*. *burgdorferi*. To this end, we fused the *ospC* promoter (P_*ospC*_) to the *luc* reporter and assessed its signal relative to light generated from *luc* linked to the constitutively expressed *flaB* promoter (P_*flaB*_). The *B*. *burgdorferi* bioluminescent reporter strains to monitor *ospC* and *flaB* will be referred to as P_*ospC*_*-luc* and P_*flaB*_*-luc* for this study. To test whether the bioluminescence driven by the P_*ospC*_ reporter construct corresponded with the known *in vitro* expression patterns for *ospC* in response to environmental changes, P_*ospC*_*-luc* was grown microaerophilically at 32°C, pH 8 or pH 7 (Fig 1) [13]. Serving as a negative control, ML23 pBBE22*luc*, referred to in this study as the P_*flaB*_*-luc* strain, was cultivated under the same conditions. As expected, P_*flaB*_*-luc* bioluminescence did not change in response to pH while the P_*ospC*_*-luc* was 4.6-fold higher at pH 7 relative to pH 8 (Fig 1A). Western analysis of the native OspC in P_*ospC*_*-luc* and P_*flaB*_*-luc B*. *burgdorferi* demonstrated higher levels of protein production at the lower pH as expected (Fig 1B). Antibody against the Firefly luciferase (Luc) protein showed an increase in Luc at pH 7 when linked to P_*ospC*_, but equal production in the P_*flaB*_*-luc* strain independent of pH demonstrating that P_*ospC*_*-luc* regulates bioluminescence and Luc production in the same manner as native *ospC*/OspC in response to an environmental cue (Fig 1B) [13].

### Temporal evaluation of *in vivo* borrelial infection and *ospC* expression

**Fig 2:**
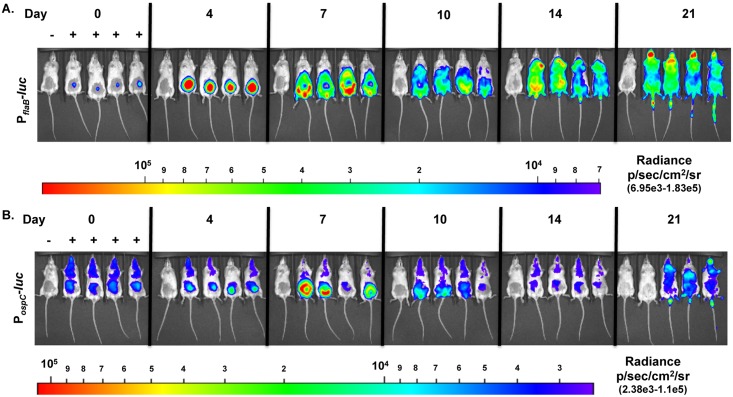
Temporal monitoring of P_*flaB*_*-luc* and P_*ospC*_*-luc* expressing *B*. *burgdorferi* during experimental infection. Balb/c mice were infected with P_*flaB*_*-luc* (A) or P_*ospC*_*-luc* (B) reporter strains at 10^5^ by ventral intradermal infection, treated with D-luciferin and imaged by IVIS at 0, 4, 7, 10, 14 and 21 days post-infection. A background control mouse was included in each group that was infected with luminescent *B*. *burgdorferi* but not treated with D-luciferin; such mouse is shown in the far left position of each image. D-luciferin treatment or the lack thereof is designated by a + or -, respectively. A 10 minute exposure was utilized to obtain images. Normalization to subtract background was performed per strain for all time points displayed in the color spectrum position under the images. P_*flaB*_*-luc* and P_*ospC*_*-luc* images are set on individual scales to display the full spectrum of bioluminescence. (A) P_*flaB*_*-luc* images were normalized to radiance range of 6.95x10^3^-1.83x10^5^ p/sec/cm^2^/sr. One-way ANOVA followed by Tukey’s Multiple Comparison test was performed to determine significant difference resulting in a *p-*value of 0.0132. A *p*-value < 0.05 is considered significant. (B) P_*ospC*_*-luc* images were normalized to radiance range of 2.38x10^3^-1.1x10^5^ p/sec/cm^2^/sr. One-way ANOVA followed by Tukey’s Multiple Comparison test was performed to determine significant difference resulting in a *p-*value of 0.0009. A *p*-value < 0.05 is considered significant.

**Fig 3:**
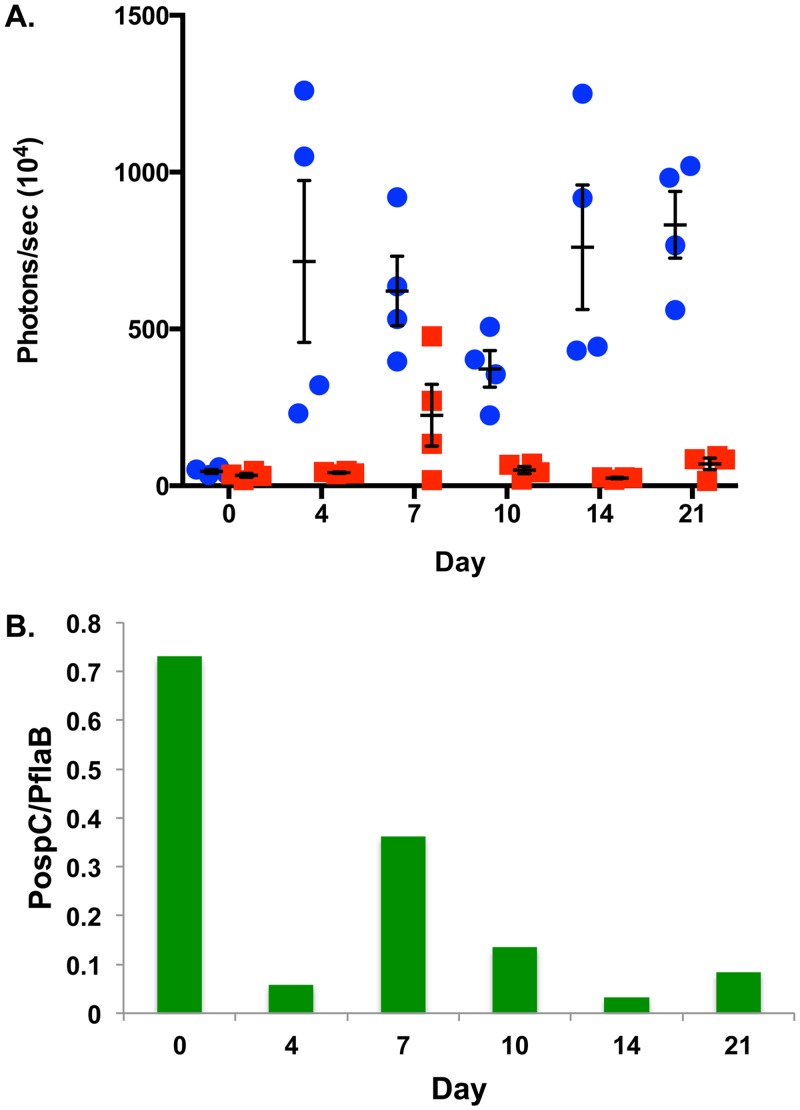
Quantitation of P_*flaB*_*-luc* and P_*ospC*_*-luc* expressing *B*. *burgdorferi* using bioluminescent readout. Five Balb/c mice were infected with 10^5^ P_*flaB*_*-luc* or P_*ospC*_*-luc* containing *B*. *burgdorferi* and 4 mice treated with D-luciferin for imaging 0, 4, 7, 10, 14, and 21 days post-inoculation for bioluminescent imaging. (A) Quantitation of 1 minute exposures was performed. At all time points the whole mouse was measured to obtain a measurement in photons/sec, representing total flux. Bioluminescence from the 4 mice treated with D-luciferin was normalized by subtracting the measurement from the no D-luciferin control and averaged. Blue circles represent P_*flaB*_*-luc* and red squares P_*ospC*_*-luc*. Error bars represent standard error. One-way ANOVA analysis resulted in statistical significance for P_*flaB*_*-luc* and P_*ospC*_*-luc* with a *p*-value of 0.0132 and 0.0262, respectively. (B) To assess the expression of *ospC* independent of changes in borrelial load, the ratio differential of P_*ospC*_*-luc* and P_*flaB*_*-luc* was calculated and represented on the y-axis. Permutation analyses comparing time points to each other found statistically significant differences (*p* < 0.05) between all comparisons, except between day 4 and day 21.

We evaluated *in vivo ospC* expression during experimental infection utilizing the P_*ospC*_*-luc* reporter strain and, based on our prior work [75], compared this to constitutively expressed P_*flaB*_*-luc* levels. As observed in previous studies, a strong localized infection developed 4 days following infection with the P_*flaB*_*-luc* strain around the site of inoculation followed by spread throughout the skin evident at day 7 and out to day 21 with fluctuations in overall emission and regions of bioluminescent intensity (Figs 2A & 3A) [75]. P_*flaB*_*-luc* radiance was significantly different (*p* = 0.0132) over the 21 days monitored indicating bacterial load fluctuates during the course of infection (Figs 2A & 3A). Bioluminescence emitted from P_*ospC*_*-luc* infected mice is substantially lower than the constitutively expressed luciferase at all time points except shortly after inoculation (Figs 2 & 3). For example, luminescence driven by P_*flaB*_ is 16.98-fold and 30.63-fold higher than P_*ospC*_ on day 4 and 14 post-inoculation, respectively (Fig 3A). P_*ospC*_-*luc* radiance is the same as P_*flaB*_-*luc* at the time of infection (Day 0) and likely represents *ospC* expression under *in vitro* cultivation conditions (Fig 3A). To accurately access *ospC* expression changes, as represented by bioluminescence, borrelial burden was taken into account by determining the ratio of P_*ospC*_-*luc* light emission relative to P_*flaB*_-*luc* bioluminescence (Fig 3B). The data show that following infection, light from the P_*ospC*_-*luc* reporter declines at day 4 relative to the overall population of *B*. *burgdorferi* as assessed by P_*flaB*_-*luc* expression. The P_*ospC*_-*luc* peaks at day 7 with 2.25x10^6^ photons/sec (p/s) representing 36% of the signal relative to P_*flaB*_-*luc*. P_*ospC*_-*luc* emits significantly lower luminescence relative to P_*flaB*_-*luc* throughout the 21 day infection with the lowest luminescence observed at day 14 coincident with possible antibody class switching (Figs 2B & 3A) [80]. The P_*ospC*_-*luc*/P_*flaB*_-*luc* ratio reaches the highest level at day 7 followed by a decrease on day 10 and 14 then increases again on day 21. Permutation analysis of the P_*ospC*_-*luc*/P_*flaB*_-*luc* values between time points indicated a statistical difference for all possible comparisons with *p-*values ranging from 0.0121 to 0.0465, except when day 4 is compared relative to day 21 (*p-*value = 0.0927). Taken together, the expression of *ospC* over an extended period, as represented by P_*ospC*_-*luc* throughout a 21 day infection, suggests that *B*. *burgdorferi* expresses this lipoprotein at distal sites, albeit at low levels within the total population. Furthermore, this spatial expression pattern suggests that OspC is needed for more than just initial colonization and, as such, may play an additional putative role in borrelial persistence.

### Quantification of bacterial load of P_*ospC*_*-luc* and P_*flaB*_*-luc* infected tissue

**Fig 4:**
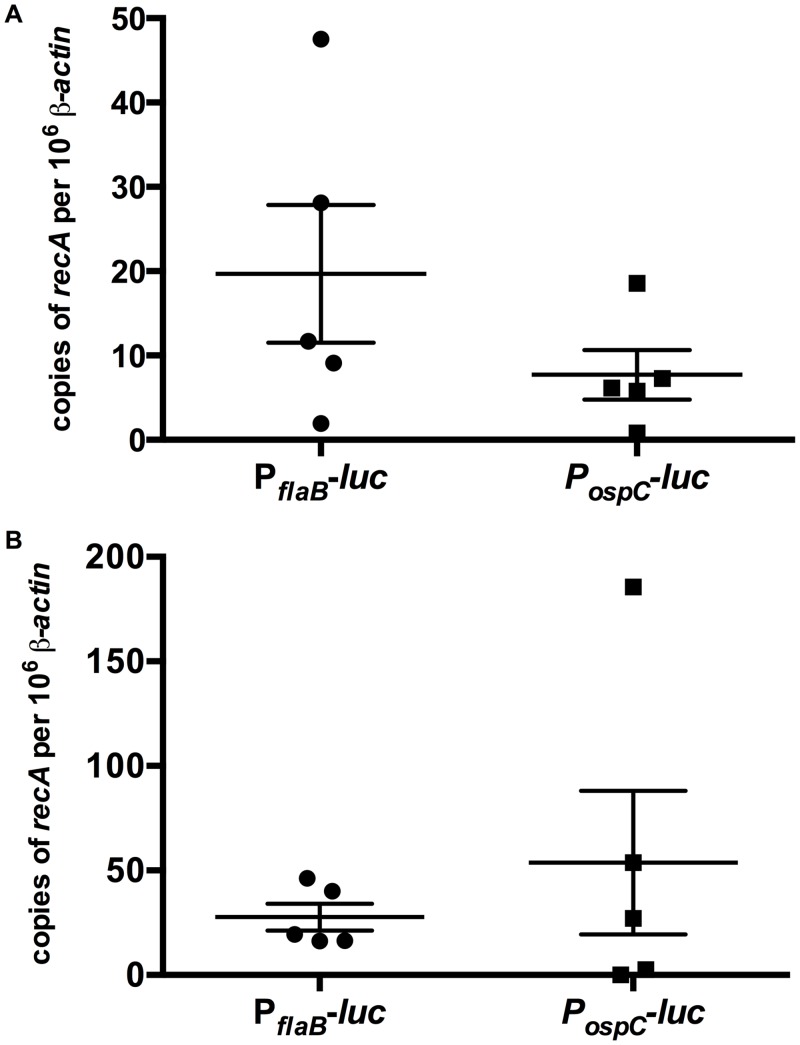
Validation of equivalent bacterial load of P_*flaB*_*-luc* and P_*ospC*_*-luc* infected Balb/c mice. Skin samples from adjacent to the inoculation site of Balb/c mice infected with 10^5^ P_*flaB*_*-luc* or P_*ospC*_*-luc* on day 10 (A) or day 21 (B) following inoculation were harvested for qPCR analysis of borrelial genomes (*recA*) per copies of 10^6^ β-actin. Horizontal bars denotes average copies of *recA* per 10^6^ β-actin and error bars represent standard error. Statistical analysis using the Mann-Whitney test indicated a lack of significance between the P_*flaB*_*-luc* and P_*ospC*_*-luc* at day 10 and 21 post-infection with *p*-values of 0.2222 and 0.9444, respectively.

To determine whether the P_*ospC*_*-luc* and P_*flaB*_*-luc* strains infected mice equivalently, flank skin samples were taken from near the inoculation site to quantify bacterial load. Total DNA from day 10 and 21 post-infection murine skin samples was evaluated for copies of *B*. *burgdorferi recA* per 10^6^ murine β-actin by qPCR (Fig 4). Bacterial loads were not significantly different between P_*ospC*_*-luc* and P_*flaB*_*-luc* skin samples at day 10 and 21 post-infection with *p-*values of 0.22 and 0.94, respectively. These results indicate P_*ospC*_*-luc* and P_*flaB*_*-luc* infected mice contain similar number of borrelial cells; therefore, differences in luminescence do not reflect a differential in *B*. *burgdorferi* presence but instead expression from the reporter utilized.

### *ospC* expression is elevated in the skin and heart during early disseminated infection

**Fig 5:**
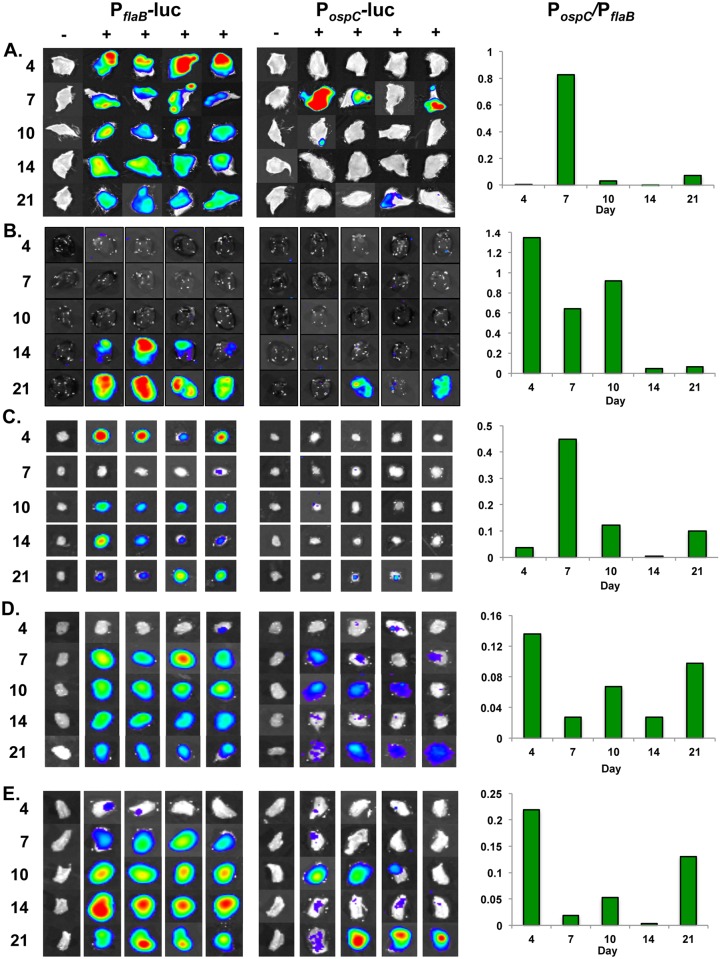
Temporal and spatial expression of *B*. *burgdorferi* containing P_*flaB*_*-luc* and P_*ospC*_*-luc* in murine tissues. Skin, inguinal lymph node, heart, bladder, and tibiotarsal joint, from P_*flaB*_*-luc* or P_*ospC*_*-luc* infected Balb/c mice were quantitatively assessed for bioluminescent emission at 4, 7, 10, 14 and 21 days post-infection. Four of the five mice were treated with a double bolus of D-luciferin and the remaining mouse served as a background control for normalization of *ex vivo* tissues. The—represents the no luciferin control and + designates the tissues treated with D-luciferin. Tissues were evaluated for bacterial load or *ospC* expression as represented by P_*flaB*_*-luc* and P_*ospC*_*-luc*, respectively. Normalization to subtract background was performed per strain for all time points displayed in the color spectrum position under the images. P_*flaB*_*-luc* and P_*ospC*_*-luc* images are set on individual scales to display the full spectrum of bioluminescence over the experimental time course. Measurable radiance above background is detectable on all days evaluated in all tissues with the exception of P_*ospC*_*-luc* infected inguinal lymph node that emits minimal luminescence. Graphs for each tissue display the ratio of P_*ospC*_*-luc/*P_*flaB*_*-luc* to depict the expression of *ospC* as measured by P_*ospC*_*-luc* relative to bacterial load (scored by P_*flaB*_*-luc*) for a given time point. The P_*ospC*_*-luc/*P_*flaB*_*-luc* ratio underwent permutation analyses comparing all time points to determine statistical significance. (A) The radiance range of skin at the site of inoculation for both strains is 4.9e3-1.83e5 p/sec/cm^2^/sr. All comparisons have a *p*-value < 0.05. (B) Heart radiance for P_*flaB*_*-luc* is 4.3e2-1.1e4 and P_*ospC*_*-luc* is 3.92e2-3.9e3 p/sec/cm^2^/sr. Two comparisons, day 4 versus day 21 and day 7 versus day 14, were not statistically significant. Remaining comparisons were significantly different (*p*-value<0.05). (C) Inguinal lymph node radiance for P_*flaB*_*-luc* is 1.1e3-1.1e4 and P_*ospC*_*-luc* is 8.32e2-1.5e3 p/sec/cm^2^/sr. Comparisons were statistically significant with *p*-values of 0.0160 or less, except day 10 versus day 21. (D) Bladder radiance for P_*flaB*_*-luc* is 1.1e3-5e4 and P_*ospC*_*-luc* is 3.78e2-1.1e4 p/sec/cm^2^/sr. There is statistical difference between early time points (day 4, 7, & 10) and late time points (day 14 & 21) with *p*-values no greater than 0.0305. (E) Tibiotarsal joint radiance for P_*flaB*_*-luc* is 1.15e3-1.15e5 and P_*ospC*_*-luc* is 4.95e2-1.1e4 p/sec/cm^2^/sr. All comparisons were statistically different (*p*-value < 0.05), except for day 4 versus day 21.

Localization of bioluminescent *B*. *burgdorferi* to track infection or evaluate gene expression has its limitations *in vivo* due to the dispersal of borrelial cells to numerous tissues and the maintenance of the spirochetes in the murine dermis that cloaks the light generated in underlying tissues inhibiting the ability to draw conclusions regarding the localization of *B*. *burgdorferi* or *ospC* expression in deeper tissues. As such, it was necessary to perform *ex vivo* imaging of specific tissues to attribute quantitative bioluminescent signal to individual sites. The evaluation of temporal and tissue specific bacterial burden and *ospC* expression was performed by *ex vivo* bioluminescence imaging and quantitation of tissues following *in vivo* assessment of mice (Fig 5 & S1 Fig). Skin, inguinal lymph node, heart, bladder, and tibiotarsal joint were harvested at day 4, 7, 10, 14, and 21 post-infection. Images were normalized to background for all time points for each tissue and strain (Fig 5).

Skin from mice infected with P_*flaB*_*-luc* reached the highest level of radiance (1.1x10^5^ p/sec/cm^2^/sr) and thus bacterial load on day 4 (Fig 5A & S1A Fig). One-way ANOVA analysis of P_*flaB*_*-luc* skin radiance indicated statistically significant changes in bacterial burden over the 21 day infection (*p* = 0.0082). P_*ospC*_*-luc* emission in skin flank peaked at day 7 with 1.1x10^4^ p/sec/cm^2^/sr and displayed the lowest luminescence on day 14, but continued to demonstrate low-level expression of *ospC* throughout the 21 day infection (Fig 5A & S1A Fig). The ratio of P_*ospC*_*-luc*/P_*flaB*_*-luc* representing *ospC* expression relative to the overall borrelial infection reached 0.82 on day 7, followed by a dramatic absence of detectable P_*ospC*_-*luc* at day 14, and partial recovery by day 21 to a ratio of 0.073 (Fig 5A). Permutation analyses, comparing all combinations P_*ospC*_*-luc*/P_*flaB*_*-luc* randomly for each time point, indicated all were significantly different across all time points with *p*-values ranging from 0.0001 to 0.0151. Taken together, these data show that the expression of *ospC* and *B*. *burgdorferi* burden in the skin varies over time and are maintained after dissemination from the inoculation site.

Previous studies have detected *ospC* transcript in the heart of mice up to two weeks following infection using endpoint analyses [37,50]. Furthermore, phage display that presented OspC peptides localized to the heart [49]. The presence of *B*. *burgdorferi* in the murine heart increases dramatically and significantly during the 21 day infection (*p* = 0.0008), specifically at day 14 and 21 relative to earlier time points as observed in P_*flaB*_*-luc* infected hearts (Fig 5B & S1B Fig). Over the course of infection P_*ospC*_*-luc* radiance increases along with P_*flaB*_*-luc* intensifying that reaches maximum bioluminescence at day 21 (S1B Fig). The higher ratio of P_*ospC*_*-luc/* P_*flaB*_*-luc* seen as 1.35, 0.65, and 0.92 is observed during day 4, 7, and 10 of infection, respectively, and are not significantly different (Fig 5B). At day 14 and 21, normalizing the P_*ospC*_*-luc* radiance for the dramatic increase of *B*. *burgdorferi* in the heart results in a ratio of 0.45 and 0.63 for P_*ospC*_*-luc/*P_*flaB*_*-luc* that is significantly lower than early time points (Fig 5B), suggesting that the previously observed increased *ospC* transcription in the heart was due to an elevated bacterial load and not increased *ospC* expression [37,50]. Our data suggest *B*. *burgdorferi* induces *ospC* expression in the heart during the first 10 days following infection, which is contrary to previously reported data that did not take into account the *B*. *burgdorferi* load within the heart [37,50].

### Inguinal lymph node colonization and induction of *ospC* expression

*B*. *burgdorferi* disseminates to the inguinal lymph node and remains colonized in this tissue throughout infection despite the presence of numerous cells involved in host immunity (Fig 5C & S1C Fig) [80,87]. Bioluminescence of P_*flaB*_*-luc* is highest in the inguinal lymph node at day 4 and 21 post-infection and signal was reduced at day 7, 10, and 14 (S1C FIg). Bacterial burden differed significantly (*p* = 0.0381) throughout the 21 day period with the most dramatic decrease in load occurring between day 4 and 7 post inoculation. P_*ospC*_-*luc* bioluminescence was the lowest in the inguinal lymph node relative to other evaluated tissues in this study, but above the threshold of detection necessary for quantitation, and remains at a similar level of radiance throughout infection. When bacterial burden is taken into account, the P_*ospC*_*-luc*/P_*flaB*_*-luc* ratio is the highest at day 7 at 0.44 due to a significant reduction of borrelial cells, but stays relatively low at the remaining time points with a P_*ospC*_*-luc*/P_*flaB*_*-luc* ratio of 0.12 or less (Fig 5C). Permutation analysis determined that the P_*ospC*_*-luc*/P_*flaB*_*-luc* ratio at day 10 and 21 was not statistically significant, but all other comparisons had a *p-*value less than or equal to 0.016. We conclude that the inguinal lymph node is readily colonized by *B*. *burgdorferi* with moderate changes in bacterial load after 7 days of infection. The low level expression of *ospC* in *B*. *burgdorferi* cells colonizing the inguinal lymph node suggesting a minimal or inhibitory role for OspC in this locale.

### Expression of *ospC* persists following secondary colonization of the bladder and tibiotarsal joint

Previous work suggests that OspC is important for early infection, but not for late infection in the murine model, while other studies suggest OspC plays a role as a dissemination facilitating factor [43–45,51,88]. We evaluated P_*ospC*_-*luc* in distal niches, e.g., the bladder and tibiotarsal joint, to determine the expression of *ospC* in these tissues. The P_*flaB*_-*luc* strain initially emits low radiance of 242.32 p/sec/cm^2^/sr in the bladder at day 4, peaks at day 7 post-infection, and then steadily declines out to day 21 with radiance of 3,799 and 1,918 p/sec/cm^2^/sr, respectively (S1D Fig). One-way ANOVA analysis of P_*flaB*_*-luc* infected bladders indicated a significant difference (*p* = 0.0056) when comparing all time points. The expression of P_*ospC*_-*luc* in the bladder is low relative to P_*flaB*_*-luc* with the highest bioluminescence observed on day 10 and 21 (S1D Fig). Normalization of bladder P_*ospC*_*-luc* expression relative to bacterial load demonstrates peaks in the overall *ospC* expression ranging from 0.07–0.14 on day 4, 10, and 21 following infection. The ratio of P_*ospC*_*-luc*/P_*flaB*_*-luc* radiance in the murine bladder is significantly different between all time points by permutation analysis, with *p*-values range ranging from 0.0126 to 0.0149, with the exception of the day 7 and day 14 comparison (*p*-value = 0.7528; Fig 5D).

Mice infected with the P_*flaB*_*-luc B*. *burgdorferi* strain reached a higher average bacterial load within tibiotarsal joints over the course of infection that differed from the bladder with the radiance increasing significantly to 20,053.1 p/sec/cm^2^/sr by day 14 (*p*<0.05), followed by a 42% reduction at day 21 (S1E Fig). Joints infected with the P_*flaB*_*-luc* strain showed significant changes in radiance during infection (*p* = 0.0009) by one-way ANOVA analysis. The murine bladder and tibiotarsal joint share a similar P_*ospC*_-*luc* bioluminescence pattern with peaks at day 4, 10, and 21 and valleys at day 7 and 14 when normalized to borrelial burden (Fig 5D & 5E). When normalized for bacterial burden, expression of *ospC* is slightly increased in the joint relative to the bladder such that a similar expression pattern is seen with ratios of 0.21 on day 4, 0.05 on day 10, and 0.13 on day 21 post-infection (Fig 5E). Tibiotarsal joint P_*ospC*_*-luc*/P_*flaB*_*-luc* is essentially the same at day 4 and 21, but statistically significantly different between the other time points with *p*-values no greater than 0.0163. Gene expression patterns of *ospC*, in the murine bladder and tibiotarsal joint, as assessed by P_*ospC*_*-luc* bioluminescence, suggest a role for this lipoprotein in later stages of infection in addition to its known role in the early infectious process [43–45].

### Correlation of *ex vivo* bioluminescence and *ospC* expression in P_*ospC*_*-luc* infected tissues

**Fig 6:**
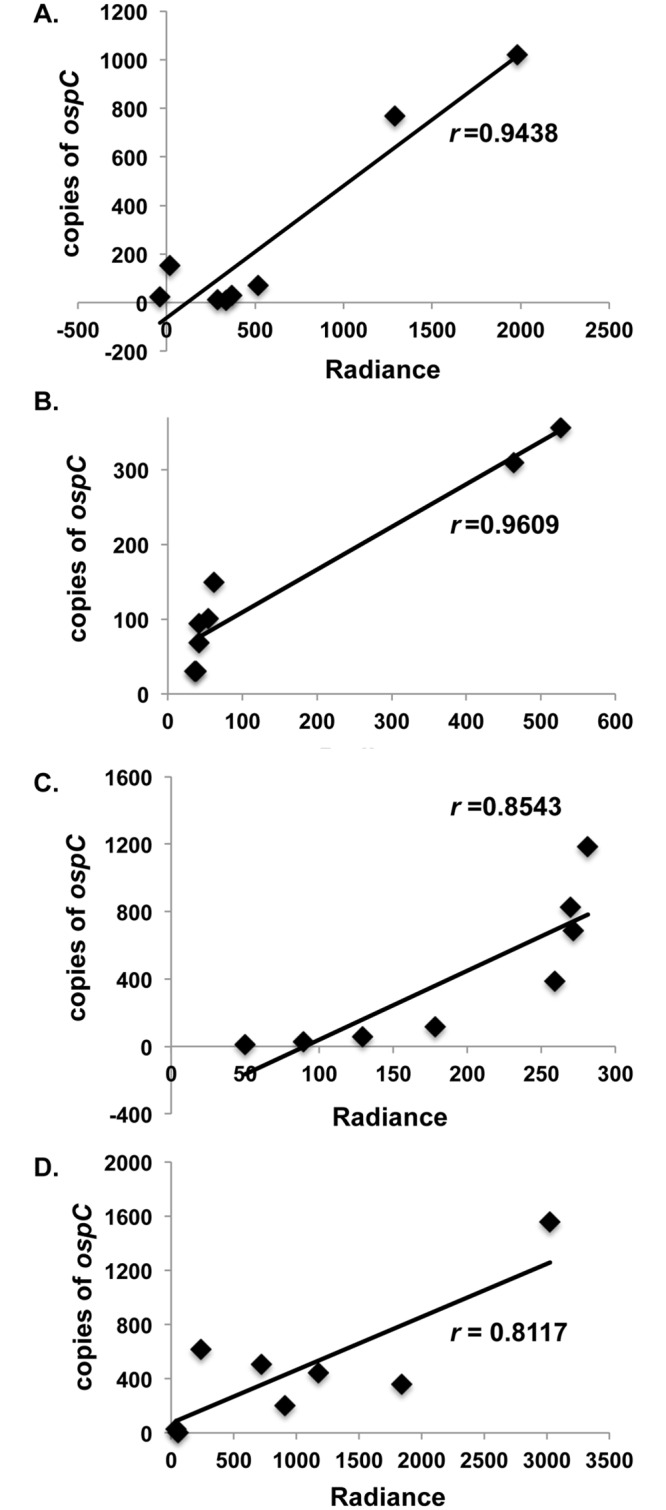
Correlation of *ospC* radiance and quantitation measure of *ospC* transcript in mammalian tissues. P_*ospC*_*-luc* infected tissues from day 10 and 21 post-infection were evaluated for radiance (p/sec/cm^2^/sr) relative to total *ospC* transcript for the whole tissue sample. Skin (A), heart (B), bladder (C), and tibiotarsal joint (D) had *r* values of 0.944, 0.961, 0.854, and 0.812, respectively.

The representation of *ospC* expression by *ex vivo* bioluminescence *B*. *burgdorferi* P_*ospC*_*-luc* reporter strain was tested to determine if the light detected correlated with native *ospC* transcript levels within mammalian tissues. Hyde et al. previously demonstrated a strong correlation between P_*flaB*_*-luc* bioluminescence and *B*. *burgdorferi* genomic copies in murine skin, suggesting that bioluminescence accurately depicts borrelial burden [75]. To determine if bioluminescence driven by P_*ospC*_ also approximates native gene expression of *ospC* in infectious *B*. *burgdorferi*, total RNA was isolated from tissue samples and analyzed by qRT-PCR. Total *ospC* transcript from tissues infected with *B*. *burgdorferi* expressing P_*ospC*_*-luc* was calculated for skin, heart, bladder and tibiotarsal joint harvested day 10 and 21 post-infection (S2 FIg). Radiance measured through *ex vivo* imaging was correlated with the total *ospC* transcript from each P_*ospC*_*-luc* infected tissue (Fig 6). All tissues displayed strong correlation between bioluminescence and quantitative molecular analysis. Specifically, the skin, heart, bladder, and joint resulted in correlation values of 0.9438, 0.9609, 0.8543, and 0.8117, respectively. The correlation of the bioluminescent P_*ospC*_*-luc* signal and *ospC* transcripts indicates that the borrelial *in vivo* P_*ospC*_*-luc* reporter accurately represents the tissue-specific expression of native *ospC* in the mammalian model and shows that the placement of the reporter construct within the shuttle vector did not disproportionally skew the bioluminescent emission spectra detected.

### *In vivo* expression of *ospC* during localized mammalian infection

**Fig 7:**
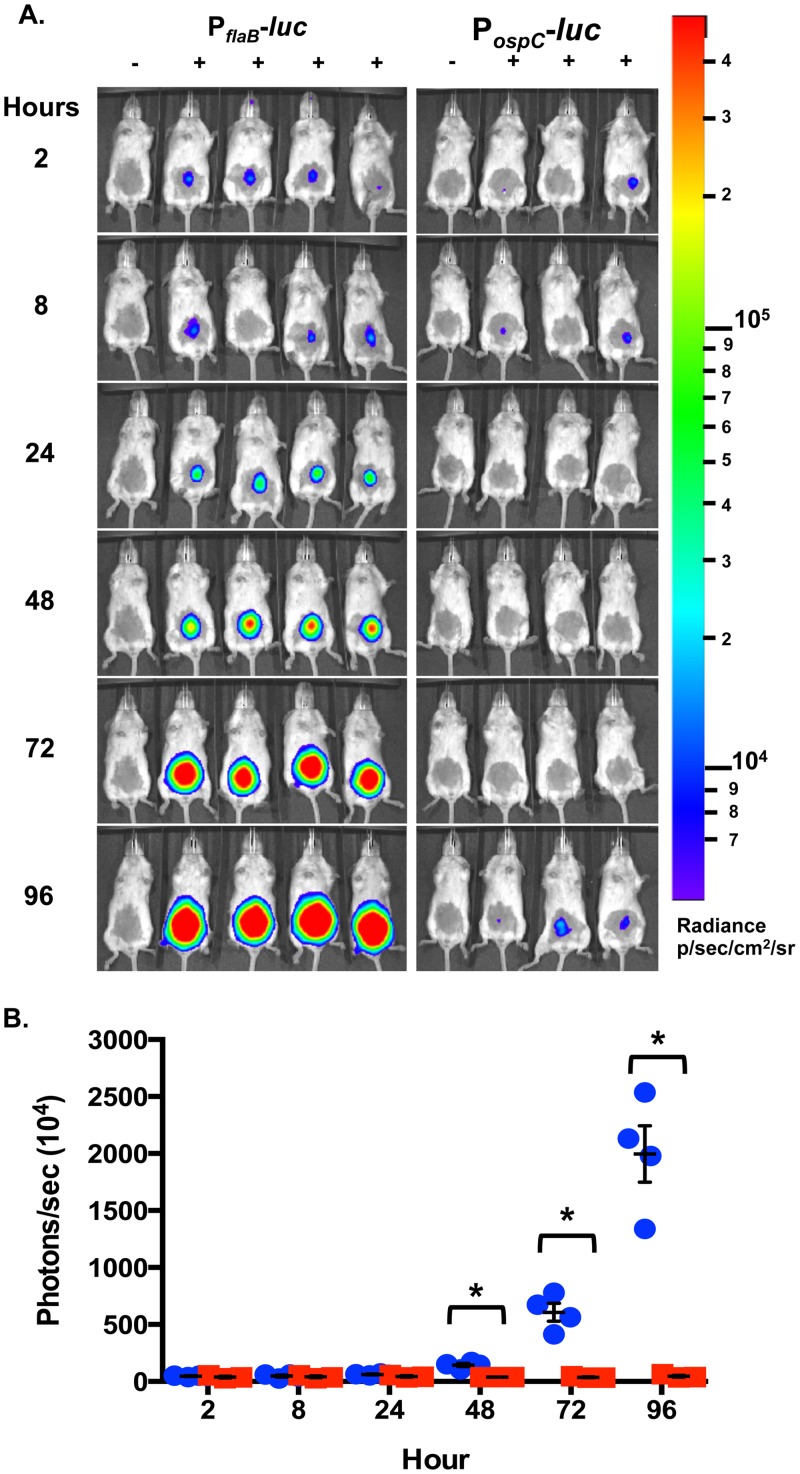
Evaluation of *ospC* expression as represented by P_*ospC*_*-luc* expressing *B*. *burgdorferi* during early infection. Balb/c mice infected by ventral intradermal injection with 10^5^ P_*ospC*_*-luc* and P_*flaB*_*-luc B*. *burgdorferi* for the quantitation of *ospC* relative to bacterial load during the 96 hours following needle inoculation. (A) Mice were treated with D-luciferin and imaged 2, 8, 24, 48, 72, and 96 hours with the exception of one no D-luciferin background control at each time point. Ten minute exposures were used to obtain images and were normalized to radiance range of 6.95x10^3^-1.83x10^5^ p/sec/cm^2^/sr. (B) Quantification of bioluminescence was determined from 1 minute exposure images. Values represent flux (photon/sec) normalized to background and the averaged value from four mice treated with D-luciferin. P_*ospC*_*-luc* is represented by red squares and P_*flaB*_*-luc* is represented by blue circles. Error bars represent standard error. An asterisk represents *p* < 0.05, indicating significant differences in bioluminescence between P_*ospC*_*-luc* and P_*flaB*_*-luc* containing *B*. *burgdorferi*. There was no significant difference in P_*ospC*_*-luc* radiance during the first 96 hours. One-way ANOVA of P_*flaB*_*-luc* radiance had a of *p*-value < 0.0001 indicating a statistically significant change in luminescence.

The role of OspC in colonization of the murine dermis has primarily been characterized with *B*. *burgdorferi* strains using *ospC* mutants to evaluate the presence of the pathogen and the associated localized immune response [44,89]. To further understand the role of *ospC* at an early stage of disease, P_*ospC*_*-luc* or P_*flaB*_*-luc* were monitored for bioluminescent emission during the first 96 hours (Fig 7). The post-infection luminescence is equally emitted from P_*ospC*_*-luc* and P_*flaB*_*-luc* infected mice out to 24 hours (Fig 7). Between 24 and 48 there is a dramatic increase in P_*flaB*_*-luc* luminescence that continues through 96 hours post-inoculation in a manner that is statistically significant by one-way ANOVA analysis (Fig 7; *p*<0.0001). Quantitation of P_*flaB*_*-luc* shows a 2.33-fold increase between 24 hour to 48 hours post-infection, indicating the bacterial load dramatically increased, but, based on the P_*ospC*_-*luc* reporter, *ospC* expression did not increase with the replication of *B*. *burgdorferi* (Fig 7B). Localized infection levels increase 9.87-fold and 32.44-fold at 72 hours and 96 hours for P_*flaB*_*-luc* expression relative to P_*ospC*_-*luc*, respectively, when compared to 24 hour luminescence levels. Differences in bioluminescence emissions between P_*flaB*_*-luc* and P_*ospC*_*-luc* were statistically significant at 48, 72, and 96 hour with *p-*values of 0.0332, 0.0350, and 0.0195, respectively. Cultivation of P_*ospC*_*-luc* and P_*flaB*_*-luc* infected skin, inguinal lymph node, and tibiotarsal joint harvested at the end point resulted in outgrowth of cells from all tissues from both groups (data not shown). Unexpectedly, P_*ospC*_*-luc* light emission showed no statistically significant change during the first 96 hours indicating that the localized expansion of *B*. *burgdorferi* does not require a corresponding upsurge in *ospC* expression.

## Discussion

The ability of *B*. *burgdorferi* to infect, disseminate, and colonize various tissues in the mammalian host is dependent on its ability to adapt to temporal pressures within specific microenvironments [4,6]. It is well known that *B*. *burgdorferi* undergoes dynamic gene regulation as it traverses through the *Ixodes* vector to the mammalian dermis, but the necessary expression of important borrelial virulence determinants in a temporal and tissue specific manner have not been fully characterized in the murine model [19]. *B*. *burgdorferi* avoids clearance by the innate immune response during early infection, hematogenously disseminates, and colonizes distal sites such as the heart, bladder, and tibiotarsal joint. Although not formally proven, it is evident that these tissues provide a permissible environment for borrelial adaptation and it is likely that unique proteins are needed to establish and maintain infection at these disparate sites [1–3]. To begin to directly address this possibility, we utilized bioluminescent *B*. *burgdorferi* to track infection in real time, as well as tissue specific expression of genes, using *ospC* as a model for *in vivo* regulation relative to the constitutively expressed *flaB* gene. Although we assumed that *ospC* expression would be restricted to early time points, our results show that the P_*ospC*_-*luc* reporter, as well as *ospC* transcripts, was detectable later in the infectious process in various tissues, suggesting a role for OspC later in the infectious process.

Lipoproteins are abundantly represented in the *B*. *burgdorferi* envelope and diversely regulated in response to environmental changes in temperature, pH, oxidative stress, CO_2_, O_2_, metals, and other yet to be identified host cues [4,6,90]. *B*. *burgdorferi* mammalian-specific virulence determinants are induced by the Rrp2-RpoN-RpoS regulatory pathway [4,25,26,36]. In response to a mammalian blood meal the response regulator, Rrp2, is phosphorylated and forms a complex with RpoN for the transcriptional activation of *rpoS* [27,28,34,38]. A member of the RpoS regulon is the surface lipoprotein OspC, which is important for the colonization of the mammalian dermis and the development of early localized disease as evidenced by the non-infectious phenotype of *ospC* mutants in both immunocompetent and immunodeficient mice [25,35,43–47,53,88]. The presence of *ospC* in *B*. *burgdorferi* is important for mammalian infection and is tightly regulated as ectopic and dysregulated overexpression of *ospC* can result in the clearance of *B*. *burgdorferi* from immunocompetent mice [44,47].

The elements potentially involved in regulation in the *ospC* promoter, -35 sequence and inverted repeats, have been examined in several studies in the context of single copy representation or multiple copies when P_*ospC*_*-ospC* is encoded on a shuttle vector with differing results [35,36,48,81]. Mutational analyses of the non-coding inverted repeats of *ospC* were examined in *trans* using *E*. *coli* as a surrogate system and directly in *B*. *burgdorferi* [35,36]. The results indicated that these sequences were not required for the regulation of *ospC*; however, their ability to affect mammalian infection was not addressed [35,36]. More recently, Drecktrah et al. performed site-directed mutagenesis of the *ospC* inverted repeats at its native locus on cp26 and found that these sequences were required for temperature and pH regulation of *ospC* [81]. Xu et al. evaluated the role of the inverted repeats during mammalian infection utilizing *trans* expression of *ospC* [48]. Here, the inverted repeats were required for repression of *ospC* and avoidance of antibody clearance in the murine model [48]. While we were restricted to the use of a P_*ospC*_*-luc* encoded on a shuttle vector, which has the potential to skew the expression pattern under cultivation conditions, Xu et al. suggest it can appropriately represent *ospC* regulation in the murine model.

Ideally, we would design these studies with P_*ospC*_*-luc* encoded in single copy so that copy number of the promoter would not represent an experimental variable, but the limitations of the technology, specifically the intensity of light emission, prevents this from being a viable option. Single copy *luc* under the control of a constitutive borrelial promoter, such as P_*flaB*_, produces substantially less light emission relative to multicopy P_*flaB*_*-luc*. Further, the single copy construct was slightly above background level and lacked the sensitivity to detect significant changes in signal intensity (Hyde, unpublished results & [91]). Further development of additional and brighter bioluminescent genes compatible with *B*. *burgdorferi* is needed to achieve this goal.

To ensure that regulation of P_*ospC*_*-luc* was faithful to the native *ospC* configuration, these operator sequences were included in the P_*ospC*_*-luc* reporter construct used in this study (Tables 1 & 2) [36,48,81]. The *B*. *burgdorferi* P_*ospC*_*-luc* strain was able to regulate bioluminescence and Luc in response to pH corresponding to that of native OspC observed in this and previous studies (Fig 1) [13,16]. The encoding of the *P*_*ospC*_*-luc* reporter cassette on a shuttle vector did not alter the expression or production patterns under the tested conditions thereby providing confidence the *P*_*ospC*_*-luc* strain would faithfully represent *in vivo* expression of *ospC* in the murine model. Furthermore, the changes in *ospC* gene expression in P_*ospC*_*-luc* infected mice observed both *in vivo* and *ex vivo*, rather than constitutive expression if regulation was not occurring, strongly supports our contention that the shuttle vector P_*ospC*_*-luc* reporter appropriately reflects the native expression of *ospC*. P_*ospC*_*-luc* bioluminescence was further validated by qRT-PCR of the *ospC* transcript (Fig 6 & S2 Fig). Here, the strong correlation seen between the P_*ospC*_*-luc* shuttle vector expression profile and native *ospC* transcript levels suggest that similar regulatory patterns exist between these constructs (Fig 6 & S2 Fig).

The function of OspC during mammalian infection has been an elusive area of research with data suggesting potential ligand binding capabilities and/or immune evasion. Specifically, OspC is a homodimer in the outer membrane that is purported to bind plasminogen, as well as the tick salivary protein, Salp15 [46,52,55,56,60,62]. While OspC may bind to host factors, its requirement during the initiation of mammalian infection suggests it plays a role in combating the innate immune response [43–45,47,48,61]. A recent study attributed an anti-phagocytic activity to OspC, whereby the clearance of *B*. *burgdorferi* lacking *ospC* by mononuclear phagocytes was substantially more than that observed for wild type *B*. *burgdorferi* [61].

The characterization of *in vivo ospC* expression at different times and in distinct niches may provide insight into the utilization of this lipoprotein by *B*. *burgdorferi*. As a part of this study we evaluated *in vivo* expression of *ospC* during the first 96 hours of infection and found that an increase in borrelial load was not accompanied by a proportional rise in *ospC* expression, suggesting limited *ospC* expression in the population is sufficient for colonization of mammalian dermal tissue (Fig 7). Following the first 96 hours of infection, P_*ospC*_*-luc* bioluminescence peaks in the skin *in vivo* and *ex vivo* 7 days post-infection indicating colonization has occurred (Figs 2, 3 and 5A & S1A Fig). It was expected that P_*ospC*_-*luc* would generate the observed levels of light emission comparable or greater than P_*flaB*_-*luc* during the initial steps of colonization considering the essential nature of *ospC* during early infection, but together these results indicate that low level *ospC* expression is sufficient in the skin to establish infection and that increased levels of expression are not observed until one week post-inoculation. A possible drawback to the study herein is the route of infection by needle inoculation rather than by tick transmission. However, this methodology ensures the inoculation of a known number of *B*. *burgdorferi*. The lack of tick transmission excludes the inhibitory activity that the salivary tick proteins afford as well as their potential effect on borrelial gene expression. The strong correlation between luminescence and quantitated copies of *ospC* transcripts in the skin, and other analyzed tissues, indicates that the P_*ospC*_-luc reporter faithfully mirrors native expression (Fig 6). It is important to note that transcription does not always translate into protein production levels and, at this time, we are unable to quantify OspC production in tissues.

Carditis is a potential complication of Lyme disease making the heart a tissue of interest in the examination of borrelial infection and pathogenesis [1]. Experiments utilizing phage-displayed OspC peptides were localized to heart and joint tissues, suggesting a potential role for OspC in dissemination and colonization within these tissues [49]. Previous studies that evaluated the expression of *B*. *burgdorferi* antigens within the heart of immunocompetent and immunodeficient C3H mice showed increased bacterial burden and *ospC* expression, particularly in the absence of the humoral immune response [47,48]. Ouyang et al. detected *ospC* transcript in the heart out to 21 days with a peak at 7 days post infection; however, no time point prior to one week were assessed [37]. In addition, Hodzic et al. observed *ospC* transcripts in the base of the murine heart out to 8 weeks with an increase in expression observed at day 7 post-infection as well [50]. However, in these studies the quantification of *ospC* transcripts was not normalized to *B*. *burgdorferi* load within a given tissue; as such, it is not possible to determine if changes in *ospC* expression could be attributed solely to changes in bacterial burden. One novel finding herein indicates that *ospC* is expressed most in the heart during the first 10 days of infection, when borrelial burden is its lowest in this tissue, thereby supporting the prior notion that OspC promotes colonization of the heart (Fig 5C) [49,50].

We also evaluated the expression of *ospC* within the inguinal lymph node. Despite their important role in host defense, *B*. *burgdorferi* readily colonizes the inguinal lymph node and remains viable throughout the course of experimental infection, as seen with the consistent detection of bioluminescence from the P_*flaB*_-*luc* construct (Fig 5B & S1B Fig). Even though borrelial cells are present, P_*ospC*_-*luc B*. *burgdorferi* emitted low level bioluminescence in inguinal lymph node throughout the 21 days of infection, suggesting that the expression of *ospC*, and presumably the production of OspC, is deleterious to *B*. *burgdorferi* in this locale (Fig 5B & S1B Fig). Recent work showed that a *B*. *burgdorferi ospC* mutant strain was able to colonize murine skin when monocytes were depleted, suggesting that OspC aids in the avoidance of phagocytic clearance during early infection [61]. Furthermore, *ospC* overexpression resulted in decreased uptake by murine macrophages. Considering the abundance of phagocytic cells that cycle through the lymph node, the lack of *ospC* expression could be interpreted as contrary to the ability of OspC to inhibit phagocytosis in macrophages [61]. However, the aforementioned study focused on the survival of *B*. *burgdorferi* solely in the skin. Macrophages and *B*. *burgdorferi* are detected in several tissues in the murine model and are thus not limited to the skin. Therefore, it would seem that OspC might be needed elsewhere to combat the ongoing assault by macrophages. Additional studies are necessary to clarify the role of OspC in this context.

To our surprise, *ospC* was expressed during later stages of the infection, particularly within the bladder and joint (Fig 5 & S1 Fig). The murine bladder and tibiotarsal joint share the most similar *ospC* expression pattern; that is, following an initial peak at day 4, a decrease is observed at day 7 and 14, with a subsequent increase again at day 21, albeit at a low level relative to the overall bacterial burden of these tissues (Fig 5). These findings are distinct from Ouyang et al. observed peak *ospC* expression in the bladder at day 7 and very little transcript out to day 21; however, this study did not take changes in borrelial burden into account [37]. Hodzic et al. was also able to detect *ospC* in joints during late stage infection (out to 8 weeks), although the number of culture positive mice and the copy number of *ospC* transcript reduced over time [50]. A possible reason for the sustained expression of *ospC* observed here could be due to joints being an immunoprotective niche that reduces the exposure of the pathogen to immune pressure [92]. Both the bladder and the joint are rich in extracellular matrices that are favored by *B*. *burgdorferi* and present potential binding ligands for several lipoproteins, including DbpA and BBK32 [75,79,84,93–95]. The increase in *ospC* expression after the colonization of distal tissues may indicate a need for OspC to maintain infection and evade the mammalian immune response. Two studies by Tilly et al. used a *B*. *burgdorferi ospC* mutant that encoded an unstable copy of *ospC*, complemented on a shuttle vector, to evaluate the requirement for *ospC* during later stages of disease [44,88]. Interestingly, their results posited that OspC is not needed later in the infectious process, following colonization and dissemination [45]. The same group demonstrated that passive transfer via the tissue transplant of a host adapted *B*. *burgdorferi*, due to the loss of the unstable shuttle vector encoding the only copy of *ospC*, resulted in positive serology in the first study and 40–67% infectivity in the second [44,88]. These studies showed the importance of *ospC* for early colonization of murine dermis, but the conflicting outcomes from the passive transfer of a borrelial *ospC* mutant are curious given the purported role for OspC in dissemination or secondary colonization. Since *B*. *burgdorferi* is a metabolically limited organism that must successfully scavenge resources from the host environment to meet basic housekeeping necessities [96], we speculate that it would not be in the best interest of *B*. *burgdorferi* to randomly express *ospC* uniquely in tissues if it was not of benefit for borrelial infectivity and resulting pathology.

Natural *B*. *burgdorferi* infection occurs with a diversity of strains and bottlenecks occur at several steps of the lifecycle, reducing the heterogeneity of the population [97,98]. A clonal borrelial infection also contains a heterogenic population with cells presenting different lipoprotein compositions during transmission from the tick midgut through the salivary glands to the mammalian dermis [99]. This heterogeneous profile likely continues throughout the spread of the pathogen and is seen by the bioluminescence data presented here in representing *ospC* expression relative to the total borrelial population. It is likely that further studies will find similar heterogeneity of other borrelial genes. Furthermore, it has been previously speculated that the bottleneck during early mammalian infection eliminates *B*. *burgdorferi* lacking *ospC* [44,45,61]. If this was the case, we would expect to observe a greater reduction of *B*. *burgdorferi* in the first few days considering that the low level expression of *ospC* does not increase with borrelial burden (Fig 6). Our work further suggests dynamic gene regulation is occurring during murine infection within a clonal *B*. *burgdorferi* population, here in the form of *ospC* expression, given that *ospC* appears to be expressed only in a fraction of the borrelial population.

## Conclusion

The data presented herein indicates that bacterial burden and gene expression of *ospC* can be evaluated in a temporal and spatial manner utilizing bioluminescent *B*. *burgdorferi* in the murine experimental model of infection. The *ex vivo* analysis allowed for a relative quantitative assessment of differential borrelial burden in murine tissues that fluctuate over time as the infection progressed. The requirement for *ospC* during early localized infection was not observed within the first few days following needle inoculation. The unique expression of *ospC* in the heart and joint, for example, using the *in vivo* bioluminescence reporter system relative to traditional qRT-PCR molecular techniques suggest different requirements for OspC to establish and maintain infection. Further studies are needed to determine the role of *ospC* and other borrelial genes in persistence. Overall, this work supports a potential additional function for OspC beyond its role in initial colonization.

## Supporting Information

S1 Fig Radiance of P_*flaB*_*-luc* and P_*ospC*_*-luc* in infected murine tissues. Bioluminescence of Balb/c tissues infected with 10^5^ P_*flaB*_*-luc* or P_*ospC*_*-luc B*. *burgdorferi* were evaluated for bacterial load and *ospC* expression, respectively. Harvested tissues were exposed for a length of time that allowed 600–60,000 counts to be obtained for quantification. Four tissues were normalized to background control tissues lacking D-luciferin treatment and averaged for radiance (p/sec/cm^2^/sr). Error bars represent standard error. The following tissues were evaluated for bacterial load (P_*flaB*_*-luc*) and *ospC* expression (P_*ospC*_*-luc*). P_*flaB*_*-luc* radiance was analyzed by one-way ANOVA to determine statistical significance and displayed in [] for each tissue. (A) skin [P = 0.0082)]; (B) heart [P = 0.0008)]; (C) inguinal lymph node [P = 0.0381)]; (D) bladder [P = 0.0056)]; and (E) tibiotarsal joint [P = 0.0009)].
(TIF)

S2 Fig Quantitation of native *ospC* transcript by qRT-PCR. Quantitative RT-PCR shows the total native *ospC* transcript of individual *B*. *burgdorferi* infected murine tissues. Four mouse skin, heart, bladder and tibiotarsal joints from day 10 (dark circles) and 21 (open squares) post-infection were evaluated for the total number of *ospC* transcripts for each tissue sample based on a standard curve. qRT-PCR for each sample and mouse was performed in triplicate and averaged. The error bars indicate standard error.
(TIF)
