# Supplementary figures and images for: In Vivo Imaging Demonstrates That Borrelia burgdorferi ospC Is Uniquely Expressed Temporally and Spatially throughout Experimental Infection
Source: PLoS One. 2016 Sep 9;11(9):e0162501. doi: 10.1371/journal.pone.0162501 (PMC5017786; doi:10.1371/journal.pone.0162501)

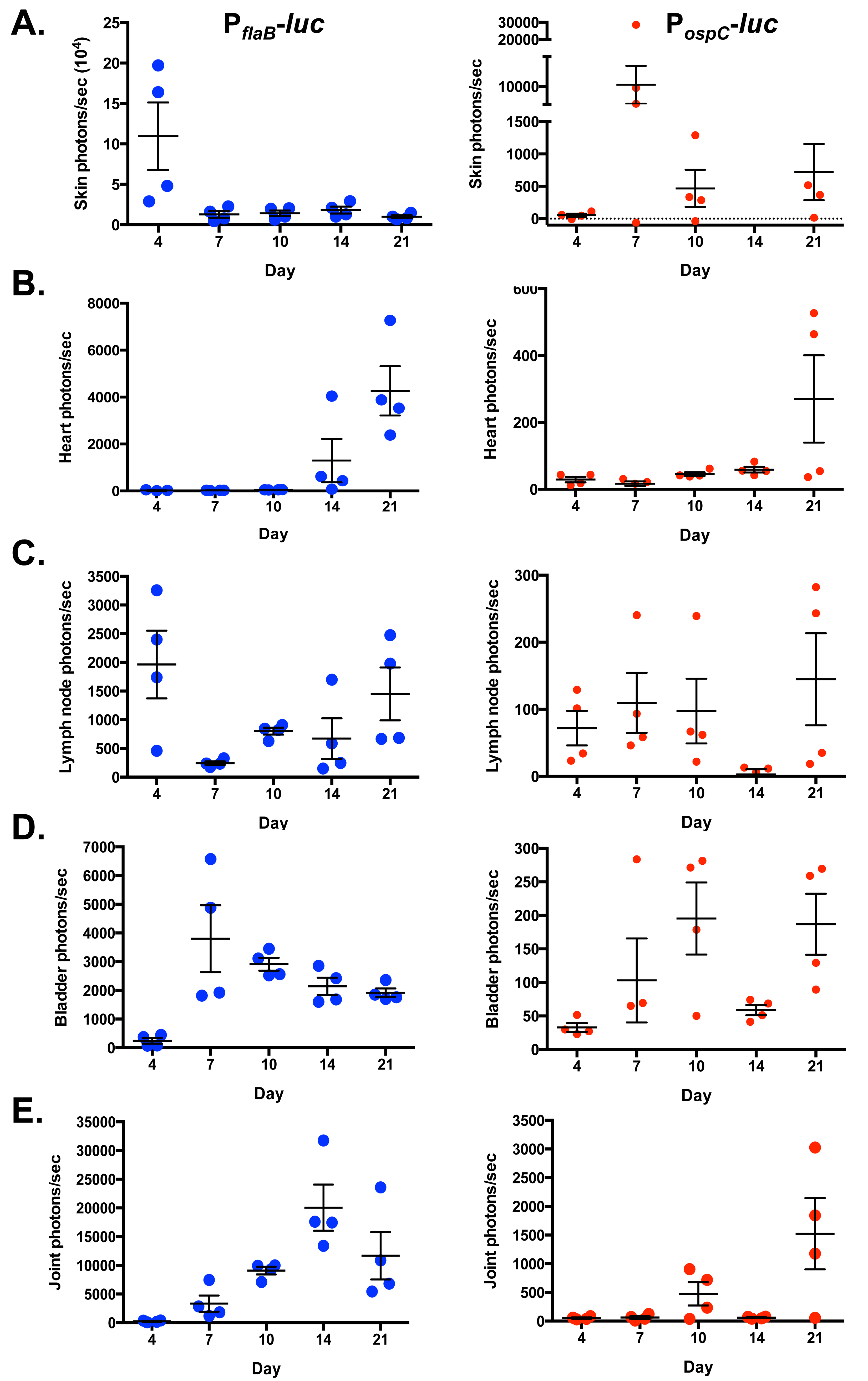

Supplement: S1 Fig — Bioluminescence of Balb/c tissues infected with 105 PflaB-luc or PospC-luc B. burgdorferi were evaluated for bacterial load and ospC expression, respectively. Harvested tissues were exposed for a length of time that allowed 600–60,000 counts to be obtained for quantification. Four tissues were normalized to background control tissues lacking D-luciferin treatment and averaged for radiance (p/sec/cm2/sr). Error bars represent standard error. The following tissues were evaluated for bacterial load (PflaB-luc) and ospC expression (PospC-luc). PflaB-luc radiance was analyzed by one-way ANOVA to determine statistical significance and displayed in [] for each tissue. (A) skin [P = 0.0082)]; (B) heart [P = 0.0008)]; (C) inguinal lymph node [P = 0.0381)]; (D) bladder [P = 0.0056)]; and (E) tibiotarsal joint [P = 0.0009)]. (TIF) [file pone.0162501.s001.tif]

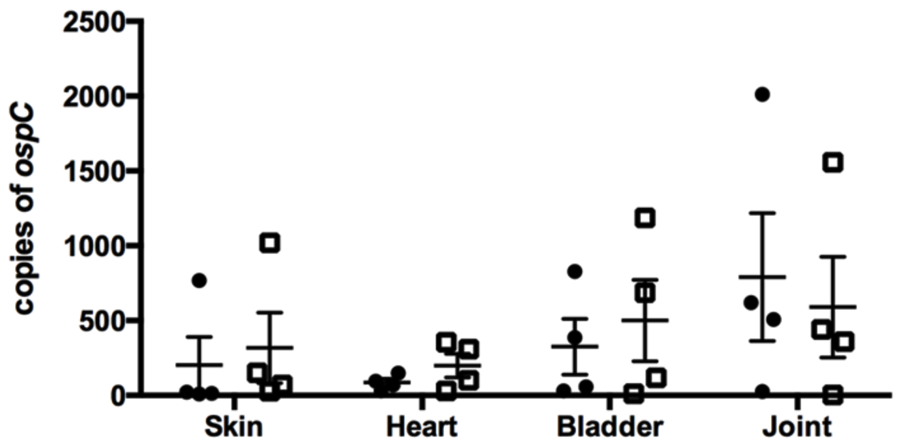

Supplement: S2 Fig — Quantitative RT-PCR shows the total native ospC transcript of individual B. burgdorferi infected murine tissues. Four mouse skin, heart, bladder and tibiotarsal joints from day 10 (dark circles) and 21 (open squares) post-infection were evaluated for the total number of ospC transcripts for each tissue sample based on a standard curve. qRT-PCR for each sample and mouse was performed in triplicate and averaged. The error bars indicate standard error. (TIF) [file pone.0162501.s002.tif]
